# Retraction: Soybean Trihelix Transcription Factors GmGT-2A and GmGT-2B Improve Plant Tolerance to Abiotic Stresses in Transgenic Arabidopsis

**DOI:** 10.1371/journal.pone.0292823

**Published:** 2023-10-09

**Authors:** The PLOS ONE Editors

Following the publication of this article [1], concerns were raised regarding multiple figures Specifically,

- In Fig 2B, the 18S rRNA panels for *GmGT-2A and GmGT-2B* appear similar.
- In Fig 3F, the following transformants appear similar:
  ○ Lane 6 in GmGT-2A(FL) row 1, Lane 4 in GmGT-2A(NT) row 2, and Lane 6 in GmGT-2B(FL) row 4.
  ○ Lane 5 in GmGT-2a(CT) row 3 and GmGT-2b(FL) row 4.
- In Fig 5A, the following panels appear to partially overlap despite representing different samples:
  ○ MS, L27 and L11
  ○ MS, L11 and L69
  ○ 125mM NaCl, WT and L69
  ○ 180mM NaCl, L11 and L69
- In Fig 5C, the following panels appear similar despite representing different samples:
  ○ 75mM NaCl, L19 and L11
  ○ 75mM NaCl, L27 and L69
  ○ 150mM NaCl, L11 and 180mM NaCl, L11
  ○ 150mM NaCl, L69 and 180mM NaCl, L69

The corresponding author stated that some of the data underlying the results in this article are no longer available.

The corresponding author acknowledged that the similarities in Fig 2B 18S rRNA panels were due to misuse of one of the panels and they provided an updated figure in which the 18S rRNA panel for GmGT-2B is replaced. They provided uncropped blots underlying the other panels in this figure, however, the original blot underlying the replacement panel was not available.

The corresponding author acknowledged that inadvertent errors may have occurred in the preparation of Fig 3F and stated that the original underlying images are not available. The editors remain concerned regarding the issues in this figure.

Regarding Fig 5A, the corresponding author stated a single plate was used for each growth condition (MS or NaCl), and they provided uncropped photos corresponding to each growth condition. Overlapping areas were adjacent to each other in the uncropped photos, however concerns remain regarding the reliability of the original data due to inconsistencies in the location of the samples between the MS and NaCl plates.

The corresponding author provided replacement images for the following samples in Fig 5C: 75mM NaCl, L11 and L69, and 150mM NaCl, L11 and L69.

In light of the concerns affecting multiple figure panels that question the validity and reliability of these data, the *PLOS ONE* Editors retract this article.

ZMX and JSZ did not agree with the retraction. HFZ, GL, WW, QYZ, CFN, YL, AGT, BM, WKZ and SYC either did not respond directly or could not be reached.
